# Editorial: Rehabilitation for somatosensory disorders

**DOI:** 10.3389/fnins.2024.1462821

**Published:** 2024-08-23

**Authors:** Yuze Zhai, Min Su, Chao Ma, Wen Wu, Fangzhou Xu, Xiaofeng Jia, Yang Zhang

**Affiliations:** ^1^Rehabilitation and Physical Therapy Department, Shandong University of Traditional Chinese Medicine Affiliated Hospital, Jinan, China; ^2^First Clinical Medical College, Shandong University of Traditional Chinese Medicine, Jinan, China; ^3^Rehabilitation Medicine Department, The Fourth Affiliated Hospital of Soochow University (Suzhou Dushu Lake Hospital), Suzhou, Jiangsu, China; ^4^Department of Rehabilitation Medicine, Sun Yat-sen Memorial Hospital, Sun Yat-sen University, Guangzhou, China; ^5^Department of Rehabilitation, Zhujiang Hospital, Southern Medical University, Guangzhou, China; ^6^International School for Optoelectronic Engineering, Qilu University of Technology (Shandong Academy of Sciences), Jinan, China; ^7^Department of Neurosurgery, University of Maryland School of Medicine, Baltimore, MD, United States; ^8^Department of Orthopedics, University of Maryland School of Medicine, Baltimore, MD, United States; ^9^Department of Anatomy and Neurobiology, University of Maryland School of Medicine, Baltimore, MD, United States; ^10^Department of Biomedical Engineering, Johns Hopkins University School of Medicine, Baltimore, MD, United States

**Keywords:** precision rehabilitation therapy, somatosensory disorders, biometric, deep learning methods for EEG biosignals, mechanisms of action

Somatosensory disorders (SD) represent a prevalent complication arising from conditions such as strokes, spinal cord injuries, and diabetic neuropathy, thereby impeding patients' ability to engage in purposeful activities and explore their surroundings (Zandvliet et al., 2020). During the initial phases of these diseases, dyskinesia tends to have a more profound impact on patients, and SD are frequently overlooked. During the generation of movements within the somatic body, the sensory system not only receives external stimuli passively but also continuously interacts and transmits information to the motor system. This interplay, either direct or indirect, profoundly influences the patient's autonomy in daily life via the somatosensory-motor pathway (Lin et al., 2022). The restoration of somatosensory function stands as a critical prerequisite for the comprehensive regain of motor capabilities (Zandvliet et al., 2020). However, given its subjective nature, SD are devoid of objective, evidence-based medical evaluation. Consequently, the treatment of SD has garnered scant attention.

Rehabilitation therapy serves as an effective adjunct to pharmacologic treatment for somatosensory disorders. Deep phenotypic assessment improves the accuracy of diagnosis and treatment of somatosensory disorders by accurately analyzing multilevel phenotypic information, solving somatosensory disorder data, and identifying comorbidities. The Research Topic “*Rehabilitation for somatosensory disorders*” encompasses eight articles, delving into aspects such as rehabilitative interventions and prognostic forecasting for SD, the relationship between SD and emotional processing and behavior in adolescents, innovative EEG biosignal deep learning methodologies, tools for detecting somatosensory acuity, as well as the mechanisms underlying transcranial direct current stimulation for fibromyalgia syndrome.

At present, the assessment of SD lacks objective criteria. Personalizing rehabilitation programs can be facilitated by identifying lesion locations and abnormal EEG patterns associated with dysfunctions (Miall et al., 2018). Hassa et al. in “*The locations of stroke lesions next to the posterior internal capsule may predict the recovery of the related proprioceptive deficits*” demonstrated that structural imaging of the internal capsule and CST can be used as a predictor of recovery from somatosensory deficits. EEG, being a non-invasive and secure neurophysiological metric, represents an auspicious avenue for the economic documentation of brain dynamics. However, the intricacies of algorithmic complexities and the impediment in realizing tailored detection stand as obstacles to the application of a self-attention mechanism for the construction of an EEG data model. In their paper titled “*EMPT: a sparsity Transformer for EEG-based motor imagery recognition*,” Liu et al. presented a pioneering neural network, EMPT, designed to decipher the temporal, spectral, and spatial features of motor imagery (MI) EEG from individuals with spinal cord injuries. The study's findings revealed that EMPT achieved an impressive 95.24% accuracy in classifying MI EEG data from spinal cord injury patients, effectively discerning EEG patterns and MI classifications in this population. Individuals with SD often adopt alternative muscle activation patterns to compensate for diminished motor function (Chen et al., 2022). Still, sustained compensatory movements may lead to secondary somatosensory impairments. Therefore, precise identification and mitigation of compensatory movements are crucial for patients with SD (Cirstea and Levin, 2000; Chen et al., 2022). In their work entitled “*Compensatory movement detection by using near-infrared spectroscopy technology based on signal improvement method*,” Chen X. et al. introduced an innovative approach demonstrating the effectiveness of NIRS signals in detecting compensatory movements and enhancing the rehabilitation outcomes for stroke patients. Moreover, somatosensory dysfunctions related to low back pain (LBP) play a role in both involuntary and voluntary postural regulation, yet there exists a scarcity of tools and methodologies for evaluating somatosensory sensitivity in this context. Notably, Chen, Tirosh et al. in their study “*Voluntary postural sway control and mobility in adults with low back pain*,” validated the reliability of the SwayDA in assessing somatosensory acuity in individuals with LBP.

Disruptions in lower limb movement and compromised somatosensory perception are intricately associated with somatic motor disorders. Emerging research indicates a prevalent occurrence of somatosensory impairments among individuals with autism spectrum disorders (ASD), and the correlations underlying these deficits have yet to be firmly established (Falcão et al., 2024). The study “*A multidimensional investigation of the relationship between skin-mediated somatosensory signals, emotion regulation and behavior problems in autistic children*” by Riquelme et al. underscores that SD can also impair the emotional processing of children and adolescents with autism. This discovery provides a theoretic groundwork for the formulation of tailored rehabilitation programs for SD.

In the domain of complementary therapies and neuromodulatory interventions, cutting-edge approaches including novel electrical nerve stimulation, intelligent rehabilitation systems, and other therapeutic modalities have demonstrated efficacy in addressing SD (Lin et al., 2024). In “*Case report: A combination of mirror therapy and magnetic stimulation to the sacral plexus relieved phantom limb pain in a patient*,” by Deng and Li, it was discovered that a synergistic approach utilizing mirror therapy and sacral plexus magnetic stimulation was capable of altering the cortical thickness in areas associated with pain perception, phantom limb, and residual limb pain. This integrated therapy notably diminished the occurrences and intensity of both phantom and residual limb pain. In “*Mechanisms of transcranial direct current stimulation (tDCS) for pain in patients with fibromyalgia syndrome*,” Wang et al. highlighted that tDCS might elicit analgesic effects by modulating neuronal activity within the brain, thus affecting cortical excitability, neurotransmission, neuroinflammation, and other related processes. SD rehabilitation endeavors to reclaim impaired neuromuscular function and restore autonomy in bodily movements. Chen, Yan et al.'s research paper “*Sensorimotor rhythm and muscle activity in patients with stroke using mobile serious games to assist upper extremity rehabilitation*” revealed that individuals employing a smartphone-based serious game to aid in rehabilitation exhibited enhanced event-related desynchronization in the region of the contralateral hemisphere associated with motion perception, along with improved therapeutic outcomes. The above research findings have improved the correctness of identifying somatosensory disorders and the stability of rehabilitation training, but it is difficult to identify individual anatomical differences, which is the direction of our future efforts.

In summation, the research conducted within this Research Topic contributes profound insights into the exact diagnosis and remediation of somatosensory ailments through the lens of precise rehabilitation. The EEG and NIRS methodologies outlined in the text are anticipated to serve as innovative evaluation instruments. Non-invasive therapeutic modalities, including magnetic and electrical stimulation, as well as rehabilitation supported by smartphone serious mobile games, are poised to represent a promising trajectory for the advancement of rehabilitation practices. Moving forward, it's important to fortify interdisciplinary collaboration across the domains of neuroscience, physiology, molecular biology, and exercise science, Further studies are conducted to validate the effectiveness and mechanism of action of the proposed method in larger scale studies. This collaborative effort aims to investigate the underlying pathogenesis of somatosensory disorders, cultivate novel diagnostic strategies, evaluative methods, and therapeutic interventions, and ultimately achieve precise, objective assessments, treatments, and prognoses for somatosensory disorders.
